# Correlation of the ratio of metastatic to non-metastatic cancer cases with the degree of socioeconomic deprivation among Texas counties

**DOI:** 10.1186/1476-072X-10-12

**Published:** 2011-02-04

**Authors:** Billy U Philips, Gordon Gong, Kristopher A Hargrave, Eric Belasco, Conrad P Lyford

**Affiliations:** 1 F. Marie Hall Institute for Rural Community Health, Texas Tech University Health Science Center, Lubbock, Texas 79430, USA; 2 Department of Agricultural and Applied Economics. Texas Tech University, Lubbock, Texas 79409, USA

## Abstract

**Background:**

Previous studies have demonstrated that cancer registrations and hospital discharge rate are closely correlated with census data-based socioeconomic deprivation indices. We hypothesized that communities with higher degrees of socioeconomic deprivation tend to have a higher ratio of metastatic to non-metastatic cancer cases (lung, breast, prostate, female genital system, colorectal cancers or all types of cancers combined). In this study, we investigate the potential link between this ratio and the Wellbeing Index (WI) among Texas counties.

**Results:**

Cancer data in 2000 were provided by the Texas Cancer Registry, while data on the ten socioeconomic variables among the 254 Texas counties in 2000 for building the WI were obtained from U.S. Census Bureau. The ten socioeconomic status variables were subjected to the principal component analysis, and the first principal component scores were grouped into deciles for the WI (1 to 10) and the 254 Texas counties were classified into 10 corresponding groups. Weighted linear regression analyses and a Cochran-Armitage trend test were performed to determine the relationship between the ratio of age-adjusted metastatic to non-metastatic cancer incidence cases and WI. The ratios of metastatic to non-metastatic cases of female genital system cancer (*r*^2 ^= 0.84, *p *= 0.0002), all-type cancers (*r*^2^= 0.73, *p *= 0.0017) and lung cancer (*r*^2^= 0.54, *p *= 0.0156) at diagnosis were positively correlated with WI.

**Conclusions:**

The ratios of metastatic to non-metastatic cases of all-type, female genital system and lung cancers at diagnosis were statistically correlated with socioeconomic deprivation. Potential mediators for the correlation warrant further investigation in order to reduce health disparities associated with socioeconomic inequality.

## Background

Socioeconomic status is one of the major determinants of health status and health disparities among different social and ethnic groups [1], and may serve as a health indicator that has predictive value in spatial epidemiologic assessment. A critical issue is how to measure the socioeconomic status at the community level using readily available census information that might be used to predict health status using information from disease registries. Variables related to socioeconomic status from census data have been used for this purpose for community assessment [2,3]. Having a reliable and easy means for performing area or community-wide assessments is useful for identifying targets for public health programs including cancer control activities. Crampton et al. developed the New Zealand Index of Relative Deprivation (NZDep91) which was constructed based on the percentages of people living in different communities meeting predefined socioeconomic deprivation criteria for nine variables derived from New Zealand census data [2]. Others have developed similar deprivation indices based on US census data. Studies have shown that these deprivation indices are good predictors of health status [3,4]. For example, Salmond et al. found that hospital discharge rate and mortality of all causes in the Wellington region and national cancer registrations for lung cancer in New Zealand were significantly and positively correlated with the NZDep91 [5]. Singh reported that US mortality of all-causes was also significantly and positively correlated with a similar deprivation index derived from US census data [4].

The study reported here was undertaken to test a specific hypothesis that the ratio of metastatic to non-metastatic cancer cases (lung, breast, prostate, female genital system, colorectal cancers or all types of cancers) would be positively correlated with the degree of socioeconomic deprivation among Texas counties. Identifying cancer in its relatively earlier non-metastatic stages leads to substantially higher success in treatment and fewer cancer deaths. Such a difference is likely to exist among communities with different levels of socioeconomic deprivation as measured by a deprivation index. Texas is the second largest state in the United States of America with 261,797 square miles, larger than France [6]. Texas has a projected population of 25,373,947 in 2010 [7]; its 77 urban counties have 80% of the population and the 177 rural counties contain the remaining 20%. Most Texas counties are classified as medically underserved with a limited infrastructure to support population health [8].

## Methods

### Data sources

This study was approved by Texas Tech University Health Science Center Institutional Review Board with exemption for review because of its use of published data. The present study used the Wellbeing Index (WI) developed by Albrecht and Ramasubramanian [3], which is derived from 10 socioeconomic status variables (Table 1) from the US census data. WI ranges from 1 to 10 with 1 as the best wellbeing or least deprivation. Thus, this index is consistent with the numeric expression of deprivation indices developed by others (also with 1 as the least deprivation) [2,4,5] and is, in fact, a deprivation index. It should be noted that the WI is constructed based on the percentages of people without cars, houses, telephones, etc. rather than the reverse percentages (of people with cars, etc.). Therefore, it may be more logical to term the WI the Index of Deprivation consistent with the New Zealand term. To avoid confusion, we use the term WI throughout the text.

**Table 1:** Variables used to build WI and the percentage of variance explained by each variable in its correlation with the first principal component (%)

Variable	%
People in households below poverty level	16.3
People over 18 without High school qualification	15.1
People in households without car	13.8
People in households without phone	11.8
People unemployed	11.6
People living in homes with too few bed rooms	9.5
People in single parent households	7.4
People with any disability	7.1
People with any form of support	6.9
People not living in own home	0.4

Data for the ten socioeconomic status variables for each of the 254 counties of Texas in 2000 were obtained from the U.S. Census Bureau [9]. Data for cancer stages were provided by the Texas Cancer Registry, Cancer Epidemiology and Surveillance Branch, Texas Department of State Health Services [10]. This database provides data by year, sex, county, etc. as well as population size for each county. We used cancer data of the year 2000 to be consistent with the 2000 US census data used to create the WI.

### Statistical analysis

The ten socioeconomic status variables listed in Table 1 were subjected to a principal component analysis (PCA) with each of the 254 Texas counties as a unit following the PRINCOMP procedure of the SAS statistical package (Cary, NC). The first principal component scores of the 254 counties were grouped into deciles for the WI (from 1 to 10) and the 254 Texas counties were classified into 10 groups corresponding to the WI. Thus, each WI group contained 25 counties with the exception of 4 groups that had the highest PCA scores where 26 counties were assigned to each group. Carcinoma in situ and localized cancers were considered as non-metastatic while cancers defined as "regional, direct extension only", "regional, regional lymph nodes only", "regional, direct extension and regional lymph nodes", "regional, NOS" and "distant" were considered as metastatic. Note that our definition of metastasis is stringent, referring to cancers that had extended to adjacent or distant organs and tissues including lymph nodes, because prognoses are often significantly different [11]. Weighted linear regression analysis (weighted by population size) and Cochran-Armitage trend test were performed with the SAS GLM and FREQ procedures, respectively, to determine the potential linear relationship between WI (explanatory continuous variable) and the ratio of metastatic to non-metastatic cancers (response variables) after adjustment for age with the 2000 U.S. standard population.

Before estimating the model, we compute the Moran's I statistic [12] and Geary's C statistic [13] in order to test for the existence of spatial autocorrelation in the regression residuals. If spatial autocorrelation is present in the residuals, then the parameter estimates are either biased or inconsistent [14]. While Moran's I test provides a general test for global spatial autocorrelation, Geary's C test is more focused and sensitive to local effects or contiguity. Both statistics are computed using the 'spdep' package in R.

In addition, we performed principal component analysis with the percentages of people with cars, etc. rather than without cars etc. to construct a "reverse" WI, and found no correlation with the ratio of metastatic to non-metastatic cases of any cancer studied, suggesting that the WI is scaling deprivation but not wellbeing.

## Results

**Figure 1:**
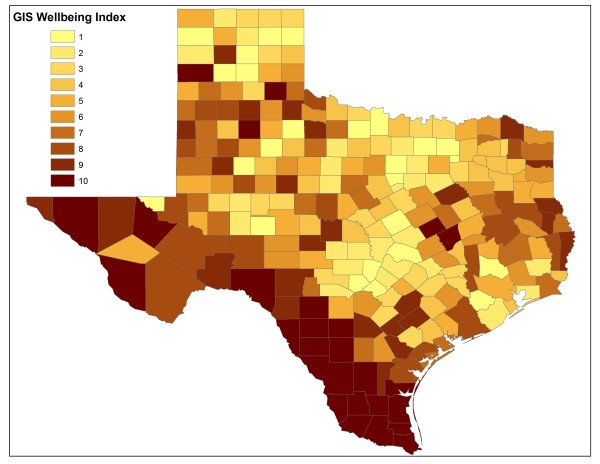
**Geographic distribution of counties with different WI in Texas in 2000**.

Table 1 presents variables that were used to build the WI and the percentages of variance explained by each variable in its correlation with the first principal component are shown. The first principal component of the ten variables accounts for 51% of the overall variance. Figure 1 shows the geographic distribution of Texas counties with different WI based on the 2000 Census information. A large proportion of counties with WI 8-10 were distributed along the U.S.-Mexico border.

**Figure 2:**
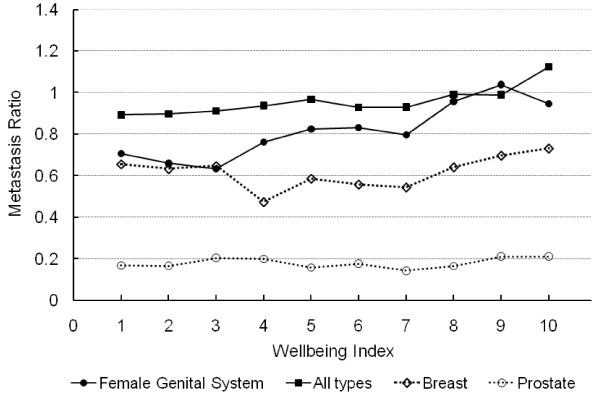
**Ratio of metastatic to non-metastatic cases of female genital system, breast, prostate and all types of cancer in relation to WI in 2000**. Statistical significance (*p *< 0.05) was observed for those cancers symbolized by solid (black) markers but not those with blank (white) symbols connected by dashed lines.

**Figure 3:**
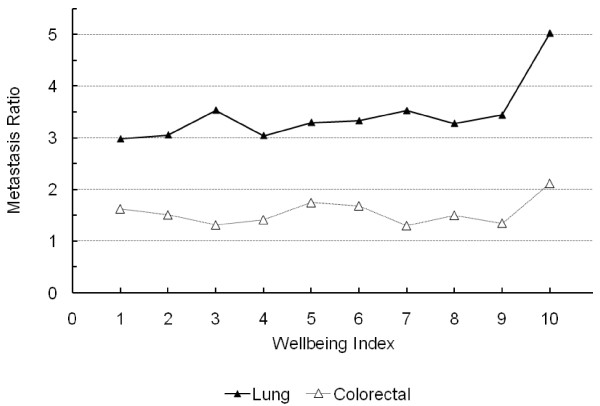
**Ratio of metastatic to non-metastatic cases of lung-bronchial and colorectal cancer in relation to WI in 2000**.

The ratio of metastatic to non-metastatic cases of the female genital system cancer (*r*^2 ^= 0.839, *p *= 0.0002), all types of cancer (*r*^2 ^= 0.728, *p *= 0.0017) and lung cancer (*r*^2 ^= 0.539, *p *= 0.0156) were significantly and positively correlated with WI (Figure 2 and 3 and Table 2). These regressions exhibit relatively high *r*-squared measures, particularly for a single linear regression model. Similar results were obtained with Cochran-Armitage trend test in terms of statistical significance (Table 2). Notably, all county congregates with WI between 4 and 7 had a ratio of metastatic to non-metastatic cancer cases of female genital system greater than that of any county congregates with WI of 1 to 3, but lower than that of those with WI of 8-10 (Figure 2). Such a linear relationship was also apparent for the metastatic to non-metastatic ratio of all types of cancer combined (Figure 2). This ratio for lung cancer is conspicuously high among counties with a WI of 10 (Figure 3). After log transformation, the linear relationship between the ratio for lung cancer and WI was still significant (*p *< 0.0268). The ratios for breast, prostate or colorectal cancers were not significantly correlated with WI (blank diamond, circle, and triangle symbols connected with dashed lines in Figure 2 and 3) (*p *> 0.05). The test for the existence of spatial autocorrelation using Moran's I and Geary's C statistics fail to detect spatial correlation in all the regressions of interest in this study and are shown in table 3. Thus, adjacency of counties had no significant effect on the ratio of metastatic to non-metastatic cancer cases, supporting the use of our regression models without the use of spatial weighting matrix to control for spatial adjacency.

**Table 2:** Correlation between Metastatic to Non-Metastatic Cancer Ratio and WI

	Weighted Linear Regression Analysis						Trend Test	
**Cancer type**	**Estimate**	**SE**	**95% CI**		***r*^*2*^**	***p***	**Z**	***p***

All Types	0.019	0.004	0.01	0.029	0.728	0.0017	-6.33	<.0001
Breast	0.005	0.008	-0.014	0.024	0.041	0.5742	-0.46	0.6564
Colorectal	0.026	0.026	-0.033	0.086	0.118	0.3311	-1.39	0.1652
F Genital	0.038	0.006	0.024	0.051	0.839	0.0002	-3.92	<.0001
Lung	0.139	0.045	0.034	0.243	0.539	0.0156	-3.18	0.0015
Prostate	0.003	0.002	-0.003	0.009	0.155	0.2608	-1.64	0.1009

**Table 3:** Tests for Spatial Correlation

	Moran's I Test		Geary's C Test	
**Cancer type**	**Statistic**	***p*-value**	**Statistic**	***p*-value**

All Types	0.733	0.232	0.808	0.210
Breast	0.154	0.439	0.995	0.160
Colorectal	1.096	0.137	0.910	0.181
F Genital	0.640	0.261	0.704	0.241
Lung	-0.160	0.564	0.783	0.217
Prostate	-1.140	0.873	-1.133	0.871

## Discussion

Previous studies have demonstrated that several aspects of health problems such as mortality of all causes, lung cancer registrations and hospital discharge rate were closely correlated with census data-based deprivation indices [2,4,5,15,16]. Results of the current study adds to the literature a new aspect of health problems in relation to socioeconomic deprivation, demonstrating for the first time that the ratios of metastatic to non-metastatic cases of all types of cancer, lung cancer and the female genital system cancer were positively and significantly correlated with the WI among Texas counties. Several factors may have mediated the correlation such as the potentially higher environmental exposure to toxins, higher rate of obesity, and racial/ethnic composition associated with socioeconomic deprivation. In addition, potentially lower rates of health insurance coverage, cancer screening, and regular checkup, difficulties in transportation, and delay in seeking medical care etc. among counties with higher degree of socioeconomic deprivation [17-20] may also be responsible for the correlation. Further investigations are warranted for the potential roles of these factors in mediating the correlation. Results from such investigations would provide crucial information for policy makers to take measures to reduce health disparities associated with socioeconomic inequality.

It should be noted that no significant correlation was found for breast, prostate and colorectal cancers. Although the exact reasons for the lack of correlation are unknown, several factors may be responsible. As pointed out above, one of the potentially significant mediators for the correlation may be lower cancer screening rates and or lower clinical checkup rate among counties with higher degree of deprivation. On the other hand, if the screening methods for certain types of cancers are not quite effective then one may or may not be able to detect significant correlation between WI and the ratio of metastatic to non-metastatic cancer cases. Recently, the U.S. Preventive Services Task Force no longer recommends screening mammography in women younger than 50 years old [21] because of high false-positive rates and low effects on mortality in spite of its widespread use [22]. Similarly, "despite widespread adoption of PSA testing, however, it remains controversial. It has been shown that elevated PSA levels do not always indicate cancer and low PSA levels do not ensure that cancer is absent [23]." Thus, screening for breast and prostate cancers may not have increased early cancer detection rate so much among counties with better WI vs. those with worse WI as to reaching a threshold for a significant correlation. Another factor that may affect the ratio of metastatic to non-metastatic cancer cases is the large percentage of aggressive breast cancer types. In a recent study on 136 consecutive female patients with suspicious breast lesions detected by mammography-ultrasound-clinical examination triple assessment and finally diagnosed with breast cancer by histological examination, 79% (128) of the 162 lesions were grade 2 or 3 (with grade 3 as the most aggressive, fast-growing type of cancer) [24]. Inflammatory breast cancer [25], anti-estrogen resistant breast cancer [26], and primary squamous cell carcinoma of the breast [27] are among the most aggressive breast cancers. As a result of the fast growing nature of this cancer, a large percentage of patients (62% in Texas as a whole) were diagnosed with breast cancer at late stages in spite of the wide utilization of screening in recent years. Since many had already developed late stage cancer with regular checkups, the potential advantage of regular checkup and screening may be compromised by the aggressiveness of this cancer.

In contrast, screening for colorectal cancer (CRC) is very effective [28]. After detection of adenomatous polyps (not carcinoma yet) by screening, polypectomy is generally performed [28]. If counties with better WI tend to have higher CRC screening rate, then two events may occur; (1) many CRC cases would be detected at early stage; (2) meanwhile, there may be a reduction in CRC cases at early stages as a result of polypectomy (otherwise polyps would have evolved to carcinoma at early stage first). Thus, polypectomy may have obscured the potential correlation between WI and the ratio of metastatic vs. non-metastatic cancer cases at diagnosis.

## Conclusions

This study demonstrates the utility of the WI as a novel tool for identifying health disparities across large geographic areas. Further, this method can be accomplished in a relatively quick and reproducible fashion using readily available and standard census data. It would be of interest to apply the method to other disease pathologies.

Specific results for cancer pathologies show that the ratios of metastatic to non-metastatic cases of all-type cancers, lung cancer and the female genital cancer at diagnosis were significantly and positively correlated to WI among Texas counties. Hence, the WI is a useful indicator at least for the ratios of metastatic to non-metastatic cancer cases at county level. Further studies are needed to determine the actual sources and solutions for cancer in economically deprived areas. In particular, barriers for the early detection of cancers among communities with higher degree of socioeconomic deprivation should be examined in order to reduce health disparities.

## Competing interests

The authors declare that they have no competing interests.

## Authors' contributions

BUP conceived the idea that the ratio of metastatic to non-metastatic cancer cases would be correlated with the WI. KAH interacted with Texas Cancer Registry and obtained the Texas cancer data. BUP, KAH and GG worked collaboratively on the data analysis. KAH performed the GIS mapping and GG performed principal component analysis, regression analysis and Cochran-Armitage trend test. BUP and GG interpreted the results and wrote the manuscript. KAH assisted GG in extracting data using SEER software, participated in the discussion of the methodology and interpretation of the results, and drew the WI map. EB and KAH used ArcGIS to obtain spatial weights followed by Moran's I and Geary's C test for spatial autocorrelation using the spdep package in R software. CL participated in revising the manuscript according to reviewers' comments, and choosing appropriate statistical approaches, and editing the entire text. All authors read and approved the final manuscript.
